# High-temperature superconductivity on the verge of a structural instability in lanthanum superhydride

**DOI:** 10.1038/s41467-021-26706-w

**Published:** 2021-11-25

**Authors:** Dan Sun, Vasily S. Minkov, Shirin Mozaffari, Ying Sun, Yanming Ma, Stella Chariton, Vitali B. Prakapenka, Mikhail I. Eremets, Luis Balicas, Fedor F. Balakirev

**Affiliations:** 1 National High Magnetic Field Laboratory, Los Alamos National Laboratory, Los Alamos, NM USA; 2 Max-Planck Institute for Chemistry, Mainz, Germany; 3 National High Magnetic Field Laboratory, Florida State University, Tallahassee, FL USA; 4 International Center for Computational Method and Software, College of Physics, Jilin University, 130012 Changchun, China; 5 State Key Laboratory of Superhard Materials, College of Physics, Jilin University, 130012 Changchun, China; 6 International Center of Future Science, Jilin University, 130012 Changchun, China; 7 Center for Advanced Radiation Sources, University of Chicago, Chicago, IL USA

**Keywords:** Superconducting properties and materials, Electronic properties and materials, Structure of solids and liquids

## Abstract

The possibility of high, room-temperature superconductivity was predicted for metallic hydrogen in the 1960s. However, metallization and superconductivity of hydrogen are yet to be unambiguously demonstrated and may require pressures as high as 5 million atmospheres. Rare earth based “superhydrides”, such as LaH_10_, can be considered as a close approximation of metallic hydrogen even though they form at moderately lower pressures. In superhydrides the predominance of H-H metallic bonds and high superconducting transition temperatures bear the hallmarks of metallic hydrogen. Still, experimental studies revealing the key factors controlling their superconductivity are scarce. Here, we report the pressure and magnetic field dependence of the superconducting order observed in LaH_10_. We determine that the high-symmetry high-temperature superconducting *Fm-3m* phase of LaH_10_ can be stabilized at substantially lower pressures than previously thought. We find a remarkable correlation between superconductivity and a structural instability indicating that lattice vibrations, responsible for the monoclinic structural distortions in LaH_10_, strongly affect the superconducting coupling.

## Abstract (web summary)

The experimental studies to understand the superconductivity of superhydrides remain scarce. Here, the authors report pressure and magnetic field dependence of superconductivity in LaH_10_, and indicate lattice vibrations strongly affect superconducting coupling.

## Introduction

For phonon-mediated superconductors, a high transition temperature necessitates light atomic masses. The lightest atom available to compose a crystal lattice is hydrogen, which forms covalently bonded molecular dimmers under ambient conditions. Transforming pure molecular hydrogen, with the aid of pressure, into a metal with an atomic lattice and into a superconductor has been a long-standing challenge and the subject of contention for the high-pressure community. Yet, chemical pre-compression with certain elements reduces the pressure required for metallization; thus, stable hydrogen-rich phases can be synthesized by the current high-pressure technology. With the discovery of a superconducting transition at the critical temperature *T*_c_ = 203 K in H_3_S at 150 GPa^1^, the search for hydrogen-rich high-temperature superconductors (HTS) has intensified, with the recent report of room-temperature superconductivity in C-S-H system with a maximum *T*_c_ of 288 K^2^. A new family of rare-earth hydrides, such as LaH_10_^3,4^ and YH_9_^5^, opened a path to a significant increase in *T*_c_, which is predicted to reach 305–326 K in YH_10_^6^.

While in H_3_S the crystal lattice is formed by H-S covalent bonds, LaH_10_ forms a clathrate-like structure, where each La atom is locked at the center of a H_32_ hydrogen cage. The interatomic distances between hydrogen atoms in LaH_10_ are close to the H–H distance predicted for atomic metallic hydrogen near *p* = 500 GPa^6^. Due to the short H–H distances and the high hydrogen content, LaH_10_ can be considered as “doped” metallic hydrogen. A pronounced isotope effect on *T*_c_ when hydrogen is substituted by its heavier isotope deuterium, confirmed that the superconductivity in HTS hydrides is induced by electron–phonon interactions^7^. However, there is a dearth of experimental studies on HTS hydrides due to the very limited number of measurement techniques available at such extreme pressures. Here we explore the superconductivity and the structure of the lanthanum hydride family over a wide range of pressures, temperatures, and magnetic fields. We find that superconductivity in LaH_10_ is strongly affected by a crystal lattice instability toward symmetry-lowering distortions. A similar dramatic change in the *T*_c_(*p*) dependence for another HTS hydride H_3_S was also linked to a structural phase transition^8–11^. The present study firmly establishes the connection between HTS and soft phonon modes that are responsible for the structural instability in hydrides.

## Results and discussion

### The relation between crystal structure and superconductivity

**Fig. 1:**
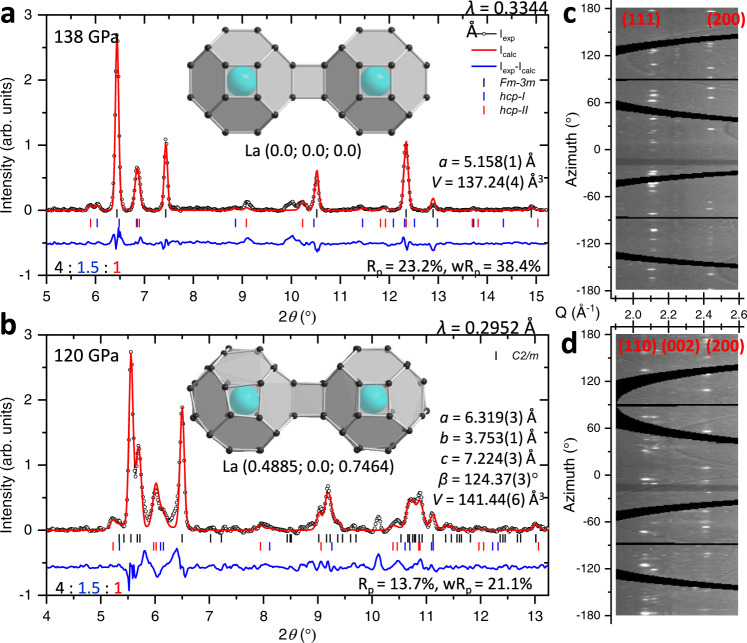
Structural data for LaH_10_ synthesized from La and excess H_2_. **a**, **b** Rietveld refinement for *Fm-3m* phase of LaH_10_ at 138 GPa and *C2/m* phase of LaH_10_ at 120 GPa, respectively. The peaks originating from the *hcp-I* (*a* = 3.668(4) Å; *c* = 5.914(11) Å; *V* = 68.9(1) Å^3^ at 138 GPa) and *hcp-II* (*a* = 3.750(3) Å; *c* = 5.561(7) Å; *V* = 67.7(1) Å^3^ at 138 GPa) impurity phases are indicated through blue and red dashes, respectively. The refined ratio between the main and the impurity phases is provided in the left bottom corner of each figure. The main structural building block, two connected LaH_32_ polyhedra, are shown in the middle inserts for each phase. Large blue and small black spheres correspond to La and H atoms, respectively. **c**, **d** The original powder X-ray diffraction patterns at 138 and 120 GPa, respectively. New reflections appear at 120 GPa due to the monoclinic distortions.

Metallic lanthanum readily reacts with hydrogen at high pressures and temperatures yielding the clathrate-like superhydride LaH_10_. We found that the superconducting *Fm-3m* phase of LaH_10_ can be synthesized at pressures much lower than ∼150–170 GPa^3,4,12,13^ as reported earlier. Specifically, the powder X-ray diffraction data show that the sample prepared in the present study under 138 GPa is comprised of the dominant *Fm-3m* phase of LaH_10_. The minor impurity phases are attributed to two hexagonal close-packed (*hcp*) phases with the *P6*_*3*_/*mmc* space group but with a different *c*/*a* ratio (∼1.63 for *hcp-I* and ∼1.48 for *hcp-II*) and a composition close to LaH_10_ (Fig. 1). Both impurity phases were also found in various samples prepared via the direct chemical reaction between hydrogen and lanthanum or lanthanum trihydride in the previous work^3^ and did not distinctly affect the *T*_c_ of the superconducting *Fm-3m* phase, which has the highest *T*_c_ in the lanthanum–hydrogen system^3^.

The persistence of the high-pressure, high-symmetry phase of LaH_10_ at pressures as low as 138 GPa corroborates recent theoretical calculations that take quantum effects into account^14^. In contrast to the classical ab initio calculations^6,14,15^, which predict structural distortions in the *Fm-3m* LaH_10_ below ∼230 GPa, the inclusion of the zero-point energy stemming from quantum atomic fluctuations lowers the enthalpy of the high-symmetry phase and stabilizes it at pressures as low as 129 GPa^14^.

The LaH_10_ sample under 138 GPa still exhibits a narrow superconducting transition toward zero resistance with a high *T*_c_ of 243 K, slightly lower than the maximum *T*_c_ of ∼250 K reported for LaH_10_ at ∼150–170 GPa^3,4,13^, in accordance with a “dome-shape” pressure dependence of *T*_c_ for the *Fm-3m* phase of LaH_10_^3^. No intrinsic hysteresis between cooling and warming *R*(*T*) curves was observed (Supplementary Fig. 1). The resistivity *ρ* of LaH_10_ is estimated to be (0.3 ± 0.1) mΩ·cm at *T* = 300 K and is higher than the value reported for H_3_S^16^. The large error bar is mainly due to the uncertainty on the thickness of the sample.

**Fig. 2:**
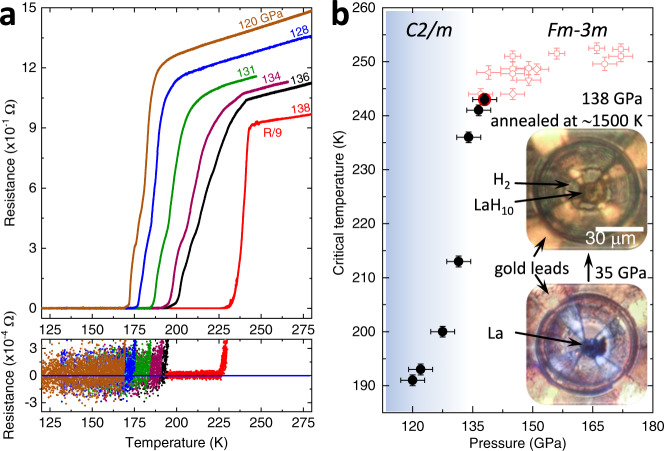
The superconducting transitions in LaH_10_. **a** The electrical resistance in LaH_10_ after the synthesis under 138 GPa (red curve), after the abrupt decompression down to 120 GPa (brown curve), and upon a gradual increase in pressure from 120 to 136 GPa (blue, green, purple, and black curves). The data measured at 138 GPa on the upper panel are divided by 9 for better presentation. **b** Pressure dependence of *T*_c_ in LaH_10_ measured in the present study (black symbols) and from a prior study^3^ (open red symbols). Insets: photos of the DAC loaded with a La flake and after the synthesis of LaH_10_ through laser-assisted heating.

After the abrupt decompression from 138 to 120 GPa, some reflections from the ancestral cubic phase became split (Fig. 1) and the *T*_c_ dropped to 191 K (Fig. 2). The powder X-ray diffraction patterns of the new distorted phase can be reasonably indexed within the *C2/m* space group (Fig. 1b). The refined cell parameters and the coordinates of the heavier La atoms are in a good agreement with theoretical models for the *C2/m* LaH_10_ phase^14,17,18^. According to the theoretical calculations^14^, the monoclinic scenario of the structural distortions is energetically more favorable than two alternative orthorhombic and rhombohedral distortions of the *Fm-3m* phase of LaH_10_ on decompression.

We found that these monoclinic structural distortions are reversible, and the high-symmetry phase can be restored if the pressure is increased. The observed *T*_c_ increases rapidly within a short pressure range with increasing pressure and reaches 241 K at 136 GPa (Fig. 2a). The broadening of the superconducting transition in Fig. 2a is likely caused by the deterioration of the phase crystallinity during variations of the pressure. The continuous change of the lattice volume during the *Fm-3m*–*C2/m* phase transition is in close agreement with both the experimental^3^ and theoretical^19^ equations of state for LaH_10_ indicating the retention of the LaH_10_ composition (Supplementary Fig. 2). In addition, predictions suggest that the composition should not change during any structural distortion scenario for the *Fm-3m* phase of LaH_10_ upon decreasing pressure^14,17,18^.

The pressure dependence of *T*_c_ in Fig. 2 displays two distinct regions—a low-pressure region characterized by a sharp rise in *T*_c_, and a high-pressure region with a much more moderate dome-like *T*_c_(*p*) dependence, with a clear boundary between the two regions at 135 GPa. This distinct shape in *T*_c_(*p*) in LaH_10_ closely resembles the *T*_c_ variation first discovered in the hydride H_3_S, where a sharp but continuous drop in *T*_c_ was attributed to the change of the crystalline structure^8,10,11^. Multiple distorted hydrogen arrangements from a high-symmetry *Fm-3m* phase are predicted for LaH_10_ as well^14^. One of the predictions reports a stable LaH_10_ *Fm-3m* phase at high pressures, with symmetric H positions and a *T*_c_ of 259 K at 170 GPa. The drop in pressure is predicted to stabilize a distorted *R-3m* phase of LaH_10_, with *T*_c_ = 206 K at 150 GPa^18^. A *T*_c_ ~ 229–245 K was calculated for the *C2/m* phase, although the calculations were performed for *p* = 200 GPa, which is substantially higher than the values presented here^17^.

### The softening of lattice vibrations

A likely explanation for the drastic change in the dependence of *T*_c_ with pressure <135 GPa is a structural phase transition in LaH_10_. The lack of a discontinuous jump in *T*_c_ in LaH_10_ and in H_3_S^8,10^ points to a continuous symmetry-lowering lattice distortion or a phase transition of the second order or weakly first order. We calculated phonon dispersion relations for the high-symmetry *Fm-3m* and distorted *C2/m* phases of LaH_10_ and identified the lattice vibrations that soften upon decompression and can be linked to the observed lattice distortion (Supplementary Fig. 4). The calculated phonon dispersions show that the *Fm-3m* phase is dynamically stable at 200 GPa. However, the softening of the low-lying H-H “wagging” vibration modes along the Γ–*Χ* direction is found in the phonon spectrum (Supplementary Figs. 4 and 5), which leads to a structural instability toward the monoclinic *C2/m* distortion. The classical harmonic treatment of atomic vibrations for the *Fm-3m* phase of LaH_10_ shows negative phonon frequencies at pressures <180 GPa.

The transformation of the crystallographic structure from a higher- to a lower-symmetry phase is governed by the phonon softening when the frequency of the collective atomic movement approaches zero. Such a drastic change in the phonon modes often has a profound effect on the phonon-mediated superconducting order. A boost in the *T*_c_ due to phonon softening in the vicinity of a structural transition has been reported in a number of superconducting families, ranging from Sn nanostructures^20^, A15 compounds^21^, intercalated graphite^22^, ternary silicides^23^, and even some elements under pressure^24,25^. The symmetry-lowering distortion in the H sub-lattice in LaH_10_ is driven by the softening of the low-lying H-H vibration modes below 500 cm^−1^ (Supplementary Figs. 4 and 5), leading to a stronger electron–phonon interaction in the *Fm-3m* phase, which is characterized by a coupling constant $\lambda =2{\int }_{0}^{\infty }{{\alpha }^{2}F(\omega )\omega }^{-1}{{{{{\rm{d}}}}}}\omega$, where $\omega$ is the phonon frequency, $F\left(\omega \right)$ is the phonon density of states, and ${\alpha }^{2}$ is an average square electron–phonon matrix element. While the light atomic mass of hydrogen is a necessary requirement for phonon-coupled HTS, *T*_c_ is also strongly affected by $\lambda$^26–29^, with a peak in *T*_c_ predicted for large $\lambda$ ~ 2–2.5, which should occur in the vicinity of the lattice instability in HTS hydrides^30^.

### Lattice distortion effect on superconducting parameters

The impact of the structural instability on the key parameters of the superconducting phase, including the upper critical field, *H*_c2_, and the superconducting coherence length, $\xi$, for the *Fm-3m* and *C2/m* phases of LaH_10_ was confirmed through magnetotransport measurements. The samples were electrically connected in a van der Pauw configuration (Fig. 2c, inset), making the measurements of both resistivity and Hall effect possible. The LaH_10_ sample under 120 GPa was measured up to 45 T in direct current (DC) magnetic fields, and the LaH_10_ sample under 136 GPa was measured in a 65 T pulsed magnet.

**Fig. 3:**
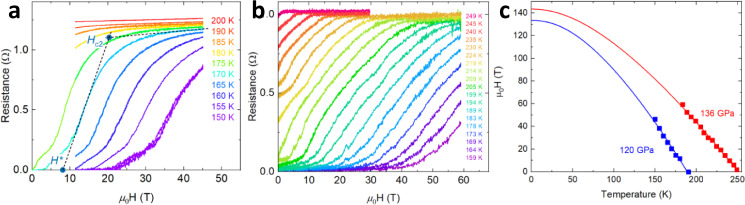
Resistance of LaH_10_ as a function magnetic field at different temperatures. **a** DC field measurements for the *C2/m* phase of LaH_10_ at 120 GPa. Two dashed lines extrapolate the slope of the high-temperature superconducting transition (left line) toward the asymptotic trace representing the high-field normal state magnetoresistance (right line) at 170 K, respectively. The intersection between two lines provides an estimation of the upper critical field (*H*_c2_). The intersection of the first line with the horizontal axis indicates the irreversibility field (H*) for the high-temperature superconducting phase. **b** Pulsed field measurements for the *Fm-3m* phase of LaH_10_ at 136 GPa. Both DC and pulsed field traces were recorded under isothermal conditions with no observation of eddy current-generated Joule heating due to the sweeping of the field. **c** Fits of the superconducting upper critical *H*_c2_ to the Werthamer–Helfand–Hohenberg (WHH) formalism. Red and blue squares denote the loci of *H*_c2_ of LaH_10_ at 136 and 120 GPa, respectively. Lines of the same color correspond to the WHH fits of the experimental data.

The magnetoresistance (MR) of LaH_10_ collected at fixed temperatures is shown in Fig. 3. Under external magnetic fields, the superconducting transitions span tens of teslas, which correlates with the broadening of the superconducting transition at zero field (Fig. 2a). The normal state MR above *H*_c2_ is nearly field and temperature independent, with a clear kink at the onset of superconductivity at *H*_c2_. For the consistency with prior studies, the *H*_c2_ values are determined as the intersection between the straight line extrapolations of the normal state MR and the slope of the superconducting transition by a method similar to the one followed in ref. ^16^. The irreversibility field of the high-temperature superconducting phase (H*) is taken by extrapolating the leading edge of the transition to the horizontal axis (Supplementary Fig. 6). The Hall resistance signal measured above *T*_c_ is consistent with the electron-like Fermi surface (Supplementary Fig. 7).

Upper critical field measurements in H_3_S HTS hydride have independently verified a large $\lambda$ ~ 2^16^. We find a substantially larger *H*_c2_ for LaH_10_ and determined that magnetic fields of the order of 100 T are required to distinguish between a strongly coupled scenario with a large $\lambda$ and the more commonly employed Werthamer–Helfand–Hohenberg (WHH) model derived in the weakly coupling limit, $\lambda \ll 1$^31^. To extract the key superconducting properties of LaH_10_ and explore the effects of the structural transition on its superconductivity, we fit the temperature dependence of *H*_c2_ to the WHH (Fig. 3c and Table 1) formalism. The WHH model fits our data well for fields up to 60 T, which is our upper measurement limit. The WHH model considers the combined effects of the magnetic field on the orbital motion and on the spin of the electrons: ${H}_{{{{{{\rm{c}}}}}}2}^{-2}={H}_{{{{{{\rm{orb}}}}}}}^{-2}+{H}_{{{{{{\rm{p}}}}}}}^{-2}$, where ${H}_{{{{{{\rm{orb}}}}}}}$ and ${H}_{{{{{{\rm{p}}}}}}}$ are the orbital-limited and spin-limited (Pauli) critical fields, respectively. We obtain ${H}_{p}$(0) values of 352 T at 120 GPa and 457 T at 136 GPa. ${H}_{{{{{{\rm{p}}}}}}}$(0) values are larger by a factor of ~3 when compared to the *H*_c2_(0) values listed in Table 1, indicating predominantly orbital-limited upper critical fields in HTS LaH_10_, which is similar to H_3_S^16^.

**Table 1:** Summary of sample properties and the associated WHH fit parameters: the critical temperature, the upper critical field at *T* = 0 K, coherence length at *T* = 0 K, BCS Fermi velocity, calculated bare-band Fermi velocity, and the slope of *H*_c2_ at the critical temperature.

Sample structure	*p* (GPa)	*T*_c_ (K)	*H*_c2_ (0) (T)	*ξ*(0) (nm)	${{{{{{{\rm{BCS}}}}}}}\;\upsilon }_{{{{{{\rm{F}}}}}}}\,$ (×10^5^ m/s)	${{{{{{{\rm{Band}}}}}}}\;\upsilon }_{{{{{{\rm{F}}}}}}}\,$ (×10^5^ m/s)	d*H*_c2_/d*T*|_*T*c_ (T/K)
*C2/m* LaH_10_	120	189	133.5	1.57	2.17	3.73	−1.12
*Fm*-*3m* LaH_10_	136	246	143.5	1.514	2.77	4.99	−0.83

**Fig. 4:**
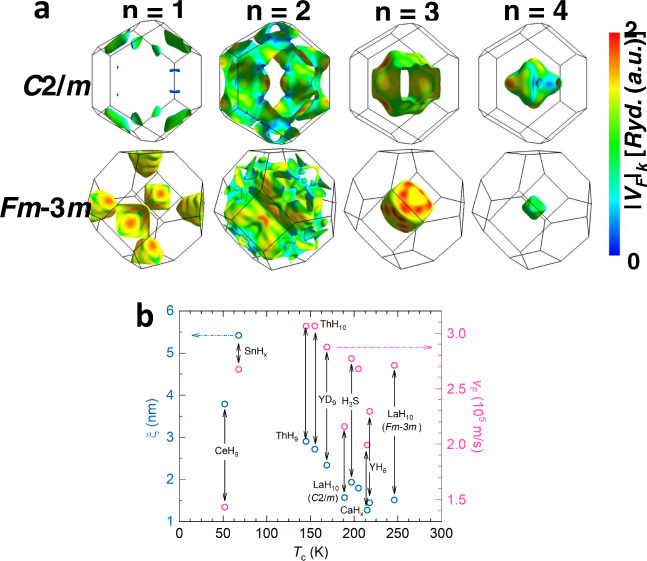
Fermi velocities for the different hydrides. **a** Calculated Fermi velocities associated with the electronic states on the Fermi surfaces in the first Brillouin zone (frame) where the color scale goes from blue (slowest) to the red (fastest). The perspective comprises angles of rotation with respect to the *x*-, *y*-, *z*-axis of 0°, 0°, and 0° for the *C2/m*-LaH_10_ and 13°, −13°, and 1° for the *Fm-3m*-LaH_10_, respectively. **b** Extracted coherence lengths ξ (cyan circles, left axis) and BCS Fermi velocities *v*_F_ (magenta circles, right axis) for the different hydrides^5,13,19,41–43^. The labels associated with the *C2/m*-LaH_10_ and *Fm-3m*-LaH_10_ phases correspond to results from the present study.

The WHH fit provides a reasonable estimate of the superconducting coherence length $\xi =\sqrt{{{{{{{\rm{\phi }}}}}}}_{0}/2{{{{{\rm{\pi }}}}}}{H}_{{{{{{\rm{c}}}}}}2}}$, where ${{{{{{\rm{\phi }}}}}}}_{0}$ is the magnetic flux quantum. There is a significant drop in *T*_c_ in the distorted phase of LaH_10_ at 120 GPa when compared to the LaH_10_ sample at 136 GPa. Surprisingly, *H*_c2_(0) only drops by a small amount and thus $\xi$ (0) remains nearly unchanged. $\xi$ is linked to both *T*_c_ and the Femi velocity ${\upsilon }_{{{{{{\rm{F}}}}}}}$: $\xi =0.18\,\hslash {\upsilon }_{{{{{{\rm{F}}}}}}}/{k}_{{{{{{\rm{B}}}}}}}{T}_{{{{{{\rm{c}}}}}}}$ within the BCS theory^7^, but the $\xi \sim {\upsilon }_{{{{{{\rm{F}}}}}}}/{T}_{{{{{{\rm{c}}}}}}}$ rule should remain valid for other models, thus signaling a lower value for ${\upsilon }_{{{{{{\rm{F}}}}}}}$ in the *C2/m* phase at 120 GPa when compared to that in the *Fm-3m* phase at 136 GPa. The onset of the lattice distortion is expected to be strongly affected by the electron dispersion, e.g. via the flattening of the bands at the boundaries of the new Brillouin zone, which may lead to a drop in ${\upsilon }_{{{{{{\rm{F}}}}}}}$ so that $\xi$ and *H*_c2_ remain high despite the drop in *T*_c_ in the *C2/m* phase. We calculated the ${\upsilon }_{{{{{{\rm{F}}}}}}}$ values along the Fermi surfaces in the first Brillouin zone for both *C2/m* and *Fm-3m* phases (Fig. 4a). The average calculated ${\upsilon }_{{{{{{\rm{F}}}}}}}$ values are listed in Table 1 alongside with the BCS values obtained from *H*_c2_(0). The calculated ${\upsilon }_{{{{{{\rm{F}}}}}}}$ values are larger than BCS ones mainly because the calculations do not account for the renormalization of the bare-band ${\upsilon }_{{{{{{\rm{F}}}}}}}$ due to electron–phonon coupling. Nevertheless, the model provides a more reliable estimate of the relative change in ${\upsilon }_{{{{{{\rm{F}}}}}}}$. The calculations confirm a ~30% drop in ${\upsilon }_{{{{{{\rm{F}}}}}}}$ in the *C2/m* phase as observed in the high-field experiments. A comparative review of the ${\upsilon }_{{{{{{\rm{F}}}}}}}$ values for other hydrogen-rich HTS families, which can be extracted from the published *H*_c2_ data, as well as the present study, reveal a surprisingly narrow distribution close to ~2.5 × 10^5^ m/s (Fig. 4b). A similar universal Fermi velocity was first noticed in the HTS cuprates, with a surprisingly similar average value of ~2.7 × 10^5^ m/s^32^. This similarity points to a renormalization of the charge carrier band dispersion both in the cuprates and in the hydrides via a strong coupling to low-lying excitations near the Fermi level, the same coupling that is commonly considered to be responsible for the high-temperature superconductivity.

In conclusion, we have measured the properties of the superconducting LaH_10_ compound as a function of pressure, temperature, and high magnetic fields. We find evidence for a pressure-induced *Fm-3m*−*C2/m* structural transition in LaH_10_ at *p*_c_ = 135 GPa, resulting in a steep but continuous decrease in *T*_c_(*p*) below *p*_c_. A likely mechanism for the structural instability is phonon softening associated with a gradual distortion of the lattice, as proposed for another HTS hydride H_3_S. We established key superconducting quantities of superhydrides under high magnetic fields, including upper critical fields and coherence lengths. We found that the drop in the Femi velocity in LaH_10_ is consistent with the distortion-induced changes in the Brillouin zone. The proximity of a peak in *T*_c_ to a symmetry-lowering structural transition, which is now experimentally established for at least two HTS hydride families, indicates that the tuning of the soft phonon modes should be viewed as one of the main pathways toward maximizing *T*_c_ in the hydrogen-rich superconductors.

## Methods

### Diamond anvil cell

The superconducting sample of LaH_10_ were synthesized in situ in a miniature diamond anvil cell (DAC) with a maximum diameter of 8.8 mm and a body length of ∼30 mm. The DAC was designed by reworking and modifying the prototype piston/locking nut design and briefly discussed in ref. ^1^. To minimize the heating effect under high magnetic fields and provide a high mechanical strength, the body of the high-pressure cell was made of a high-purity Cu–Ti alloy with 3 wt % Ti and Cu–Be alloy with 1.8–2.0 wt % Be. These DACs allow one to reach high pressures up to 200 GPa, perfectly fit the narrow bore of high field DC and pulsed magnets, and still have wide opening-angle appropriate to the routine X-ray diffraction and spectroscopic measurements.

### Sample preparation

For the sample synthesis, a small piece of metallic lanthanum (Alfa Aesar, 99.9%) with a lateral dimension of about 10 μm and a thickness of ∼1–2 μm was placed in the center of the beveled diamond anvil with a culet size of 35 μm onto the tips of four sputtered leads. The 120-nm-thick tantalum leads covered by a 50-nm-thick gold layer were sputtered onto the diamond surface in a van der Pauw configuration with a distance of ∼4 μm between tips (see Supplementary Fig. 7 inserts). The electrical leads were thoroughly isolated from the metal rhenium gasket by a protecting layer made from magnesium oxide, calcium fluoride, and epoxy glue mixture. Excess hydrogen (Westfalen, 99.999%) was introduced in the DAC at a gas pressure of about 150 MPa and served as both a reactant and a pressure-transmitting medium. After the cell was thoroughly clamped, the sample was pressurized to a pressure of 138 GPa and then heated up to ∼1500–2000 K by a microsecond pulse YAG laser to initiate the chemical reaction between reactants. Hydrogen was always in excess, and its presence in the DAC throughout the experiment was monitored visually and by Raman spectroscopy. The pressure was estimated from the Raman shift of the diamond line edge^33^ and the vibron of H_2_^34^. Although both scales indicated the same pressure values within an error of ±5 GPa, we used the hydrogen scale throughout the manuscript.

After electrical transport and X-ray diffraction measurements for the sample of the *Fm-3m* LaH_10_ at 138 GPa, the pressure in the DAC abruptly dropped to 120 GPa during transportation. Then the pressure was increased stepwise up to 136 GPa. Zero field electrical transport properties were measured at each pressure step. X-ray diffraction data were collected at 138 and 120 GPa and magnetotransport measurements under external magnetic fields were done at 120 and 136 GPa.

### Structure characterization

X-ray diffraction data were collected at the beamline 13-IDD at GSECARS, Advanced Photon Source using *λ*_1_ = 0.2952 Å and *λ*_2_ = 0.3344 Å, beam spot size of ∼3 × 3 μm, and Pilatus 1 M CdTe detector. Typical exposure time varied between 10 and 300 s. Processing and integration of the powder X-ray diffraction patterns were carried out using the Dioptas software^35^. Indexing and Rietveld refinement were performed in GSAS and EXPGUI packages^36,37^. The coordinates of the heavier lanthanum atoms were refined from the experimental data, whereas H atoms were placed in the theoretically calculated positions. With the lattice parameters and La atom positions fixed, structural relaxation for H atom positions was carried out using the Quantum-ESPRESSO package^38^. Structural data for the refined *Fm-3m* and *C2/m* phases of LaH_10_ can be obtained as Crystallographic Information Files from the Cambridge Crystallographic Data Centre via www.ccdc.cam.ac.uk/data_request/cif, on quoting the Deposition Number: 2033292–2033293.

The present structural data contradict the antecedent experimental work^12^, in which the *Fm-3m* phase of LaH_10_ was studied upon decompression from 169 to 27 GPa. Geballe et al. found a phase transition that breaks the face-centered cubic symmetry of the crystal lattice at pressures of 152–121 GPa and a subsequent decomposition of LaH_10_ compound at lower pressures. The authors proposed an *R-3m* structural model for the distorted phase of LaH_10_, though the observed peaks in powder X-ray diffraction patterns did not match the calculated positions for this model. The same inconsistency between the observed and calculated peaks can be seen if one fits the present X-ray diffraction data at 120 GPa within the *R-3m* model (Supplementary Fig. 2), which indicates that the suggested *R-3m* model is erroneous.

We also found that the instability of the *Fm-3m* LaH_10_ phase occurs at much lower pressures than was claimed by Geballe et al.^12^ This discrepancy likely stems from the different pressure scales, which were used for estimation of the pressure values in samples. The hydrogen scale used in the present study is more accurate in comparison with the diamond scale, which is based on the stresses in diamonds and strongly depends on the arrangement of the experiment. Hydrogen is very soft even in the solid state and uniformly transmits pressure over the sample. Therefore, the peak of the hydrogen vibron is sharp and the corresponding pressure values can be determined with accuracy greater than ±3 GPa. The uncertainty of the estimation of pressure values in DAC using the diamond scale is shown in Supplementary Fig. 3b. Based on the spectroscopic study of 21 various samples of D_2_, the dispersion of the pressure values estimated using the diamond scale for the same positions of the high-wavenumber D_2_ vibron can be as high as 25 GPa in the pressure range of 100–200 GPa. The similar difference of ∼18 GPa is observed between the present data and data from ref. ^12^ on the pressure dependence of the volume per La atom in LaH_10_. Our data are in very close agreement with the equation of state of LaH_10_ calculated using the Quantum Espresso pseudopotentials^19^. Thus, the overestimated pressure value of 152 GPa, which was claimed as the beginning of the structural instability of the *Fm-3m* phase of LaH_10_ in ref. ^12^ should be reduced to ∼134 GPa, which is in perfect agreement with *p*_c_ = 135 GPa found in the present study.

### Zero field electrical transport measurements

Zero field electrical resistance was measured through a four-probe technique in van der Pauw geometry with currents ranging from 10^−4^ A at *p* = 138 GPa to 10^−3^ A at *p* = 120–136 GPa samples. No apparent effect of the current value on the measured *T*_c_ was observed. The electrical measurements are presented in a warming part of a thermal cycle as it yields a more accurate temperature reading: the sample is warmed up slowly (0.2 K/min) under nearly isothermal environmental conditions (no coolant flow). The temperature was measured by a Si diode thermometer attached to the DAC with an accuracy of ∼1 K. *T*_c_ was determined at the offset of superconductivity—at the point of apparent deviation in the temperature dependence of the resistance from the normal metallic behavior.

### Magnetotransport measurements

MR and Hall effect measurements under high magnetic fields were conducted in the 45 T hybrid magnet and in the 65 T pulsed magnet at the National High Magnetic Field Laboratory. A copper thermal shield was placed around the DAC during DC field measurements. The thermal shield was heated uniformly to reduce the thermal gradients, and a secondary Cernox thermometer was attached to the DAC gasket for accurate measurements of the sample temperature. There is no observable heating from the ramping of the magnetic field at rates up to 3 T/min. The Hall effect was measured for the sample at 120 GPa above *T*_c_ in the hybrid DC magnet from 11.5 to 45 T. Reverse-field reciprocity method was employed to determine Hall resistance ${R}_{{xy}}$^39^ because the field direction of the hybrid magnet cannot be reversed during the day shift. A high-frequency (290 kHz) lock-in amplifier technique was employed to measure sample MR in 65 T pulsed magnet. AC current 500 µA was applied to the sample, and the voltage drop across the sample was amplified by an instrumentation amplifier and detected by a lock-in. No sample heating was observed during ~50 ms long magnet pulse based on comparisons of up sweep and down sweep resistance traces at different field sweep rates.

### Computational details

Structural relaxation, electronic structures, and phonon calculations were carried out within the framework of density functional theory (DFT) as implemented in the Quantum-ESPRESSO package^38^. Structure relaxations were performed using DFT using the Perdew–Burke–Ernzerhof generalized gradient approximation^40^. Phonon dispersion calculations were performed with the density functional perturbation theory. Ultrasoft pseudopotentials for La and H were used with a kinetic energy cutoff of 80 Ry. To reliably calculate the phonon dispersion, we have employed dense ***k***-meshes and ***q***-meshes for all the phonon calculations: 8 × 8 × 4 ***k***-meshes and 4 × 4 × 2 ***q***-meshes for the *C*2/*m*-LaH_10_ structure and 12 × 12 × 12 ***k***-meshes and 6 × 6 × 6 ***q***-meshes for the *Fm-3m*-LaH_10_ structure. The visualization of the atomic vibrations was done by using a visualization tool (http://henriquemiranda.github.io/phononwebsite/phonon.html). For visualization in three-dimensional of the Fermi velocities associated with the electronic states on the Fermi surfaces in the first Brillouin zone, we have used FermiSurfer open software drawing code (http://fermisurfer.osdn.jp/).

### WHH model

Numerical fit to the WHH model for the temperature dependence of *H*_c2_(*T*) defined by orbital and spin-paramagnetic effects in the dirty limit is given by WHH^31^:

1 $${{{{{\rm{ln}}}}}}\left(\frac{1}{t}\right)=\mathop{\sum }_{\nu =-\infty }^{\infty}\left\{\frac{1}{\left|2\nu +1\right|}-{\left[\left|2\nu +1\right|+\frac{{\bar{h}}}{t}+\frac{{(\alpha \bar{h}/t)}^{2}}{\left|2\nu +1\right|+({\bar{h}}+{\lambda }_{{{{{{\rm{SO}}}}}}})/t}\right]}^{-1}\right\}$$

where $\bar{h}$ = (4/*π*^2^)[*H*_c2_(*T*)/*T*_c_(−d*H*_c2_/d*T*)_*T*c_], *α* is the Maki parameter, and *λ*_SO_ is the spin–orbit constant. The Maki parameter for each sample is estimated from the slope of *H*_c2_(*T*) at *T* = *T*_c_: $\alpha =\sqrt{2}\,{H}_{{{{{{{{\rm{c}}}}}}\; {{{{{\rm{orb}}}}}}}}}/{H}_{{{{{{{{\rm{c}}}}}}\; {{{{{\rm{p}}}}}}}}} \sim {\left.-0.52758{{{{{\rm{d}}}}}}{H}_{{{{{{\rm{c}}}}}}2}/{{{{{{\rm{d}}}}}}T}\right|}_{{T_{{{{{\rm{c}}}}}}}}$^26^.

## Supplementary information

Supplementary Information

Peer Review File

## Data Availability

The data that support the findings of this study are available in Open Science Framework with the identifier: 10.17605/OSF.IO/RUJWA. Source data are provided with this paper.

## Source data

Source Data
