# Supplementary material for: High-temperature superconductivity on the verge of a structural instability in lanthanum superhydride
Source: Nat Commun. 2021 Nov 25;12:6863. doi: 10.1038/s41467-021-26706-w (PMC8617267; doi:10.1038/s41467-021-26706-w)
Supplement: Supplementary file 1 — Supplementary Information [file 41467_2021_26706_MOESM1_ESM.pdf]

# Supplementary Information for

# "High-temperature superconductivity on the verge of structural instability in lanthanum superhydride"

Dan Sun, Vasily S. Minkov, Shirin Mozaffari, Ying Sun, Yanming Ma, Stella Chariton, Vitali B. Prakapenka, Mikhail I. Eremets, Luis Balicas & Fedor F. Balakirev

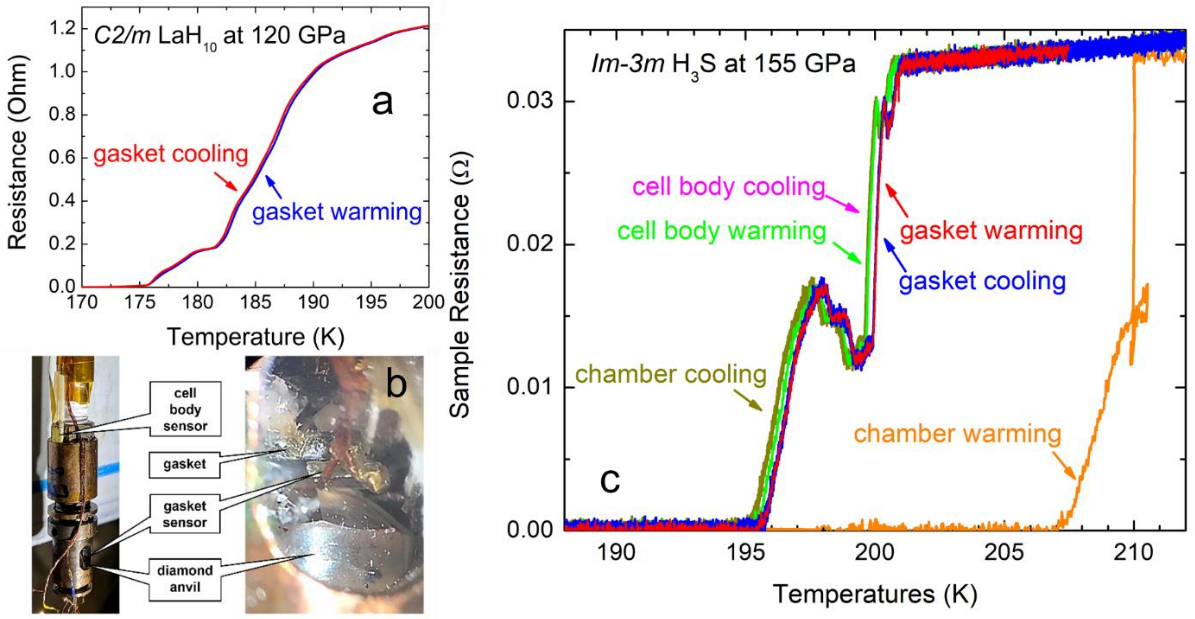


**Supplementary Fig. 1. Thermal lag in the resistivity measurements. a**, Temperature dependence of the resistivity in the $LaH_{10}$ sample at 120 GPa showing the superconducting transition during the cooling and warming cycles at zero field. There is a small shift < 0.3 K between the cooling and warming curves if the thermometer is attached to the gasket. The 0.3 K thermal lag is due to a less than ideal thermal link between the thermometer and $LaH_{10}$ sample due to a layer of protective epoxy between the thermometer and the gasket in this particular measurement. **b**, A dedicated study of the origin of the hysteresis based on the raw data from a prior publication[1]. The thermal lag was carefully characterized in a similar DAC containing the sample with the superconducting *Im-3m* phase of $H_3S$ at 155 GPa. The study was prompted by the apparent hysteresis present in the earlier HTS hydride reports and the claims that the hysteresis is intrinsic to the superconducting transition in HTS hydrides[2]. We placed multiple thermometers inside and around the DAC. One thermometer measured the temperature of the sample chamber (Oxford Instruments Variable Temperature Insert, not shown on the picture), the second thermometer (#2) was mounted on the metal body of the cell (Lakeshore Cernox SD package ~ 2 mm wide), and the third thermometer (#3) was attached directly to the gasket surrounding the sample (Lakeshore Cernox bare chip ~ 0.75 mm wide). Given that the metal gasket has a very small thermal mass and envelops the $H_3S$ sample, the bare chip thermometer #3 most effectively measures the true temperature of the $H_3S$ sample. **c**, The hysteresis between cooling and warming resistivity traces in $H_3S$ sample depends on the placement of thermometer. We find no evident hysteresis when we use gasket temperature readings. The sensor #2 attached to the metal body of the DAC always displays hysteresis, while its cooling trace matches the sensor #3.

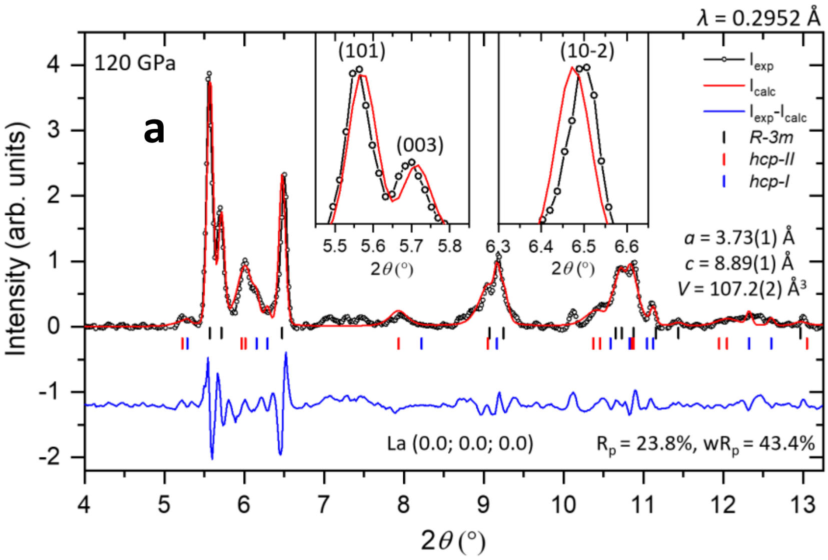


**Supplementary Fig. 2. Alternative Rietveld refinements**. **a**, Rietveld refinement for the *R-3m* model of $LaH_{10}$ at 120 GPa, which leads to a worse fit of the experimental powder X-ray diffraction pattern in comparison with the *C2/m* structural model. Insets: pronounced shifts between the calculated positions for the first three (101), (003), and (10-2) reflections in the *R-3m* structural model with respect to the observed peaks. The fitting factors resulting from the Rietveld refinement within the *R-3m* model ($R_p$=23.8% and $wR_p$=43.4%) are considerably worse than those for the *C2/m* model ($R_p$=13.7%, $wR_p$=21.1%).

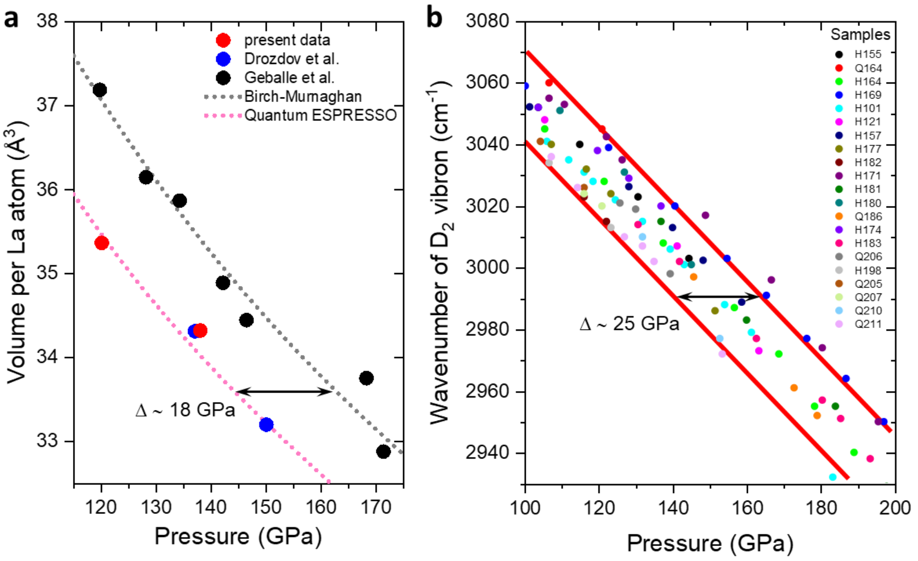


**Supplementary Fig. 3 Uncertainty on the estimation of the pressure within a DAC using different pressure scales**. **a**, pressure dependence of the volume per La atom in the $LaH_{10}$ compound. Red and blue circles correspond to the present data and data from Ref. 3, where the samples of $LaH_{10}$ were surrounded by the pressure-transmitting medium (excess $H_2$) and pressure values determined using the hydrogen scale[4]. Magenta dotted curve represents the equation of state of $LaH_{10}$ calculated using the Quantum Espresso pseudopotentials[5]. Black circles are the data form Ref . 6, where the values of pressure were evaluated using the diamond scale and the equation of state of tungsten used as the gasket material. **b**, pressure dependence of the high-wavenumber $D_2$ vibron measured for 21 different samples of pure $D_2$ within DACs (our unpublished data). The pressure values on the x-axis are estimated using the diamond scale[7].

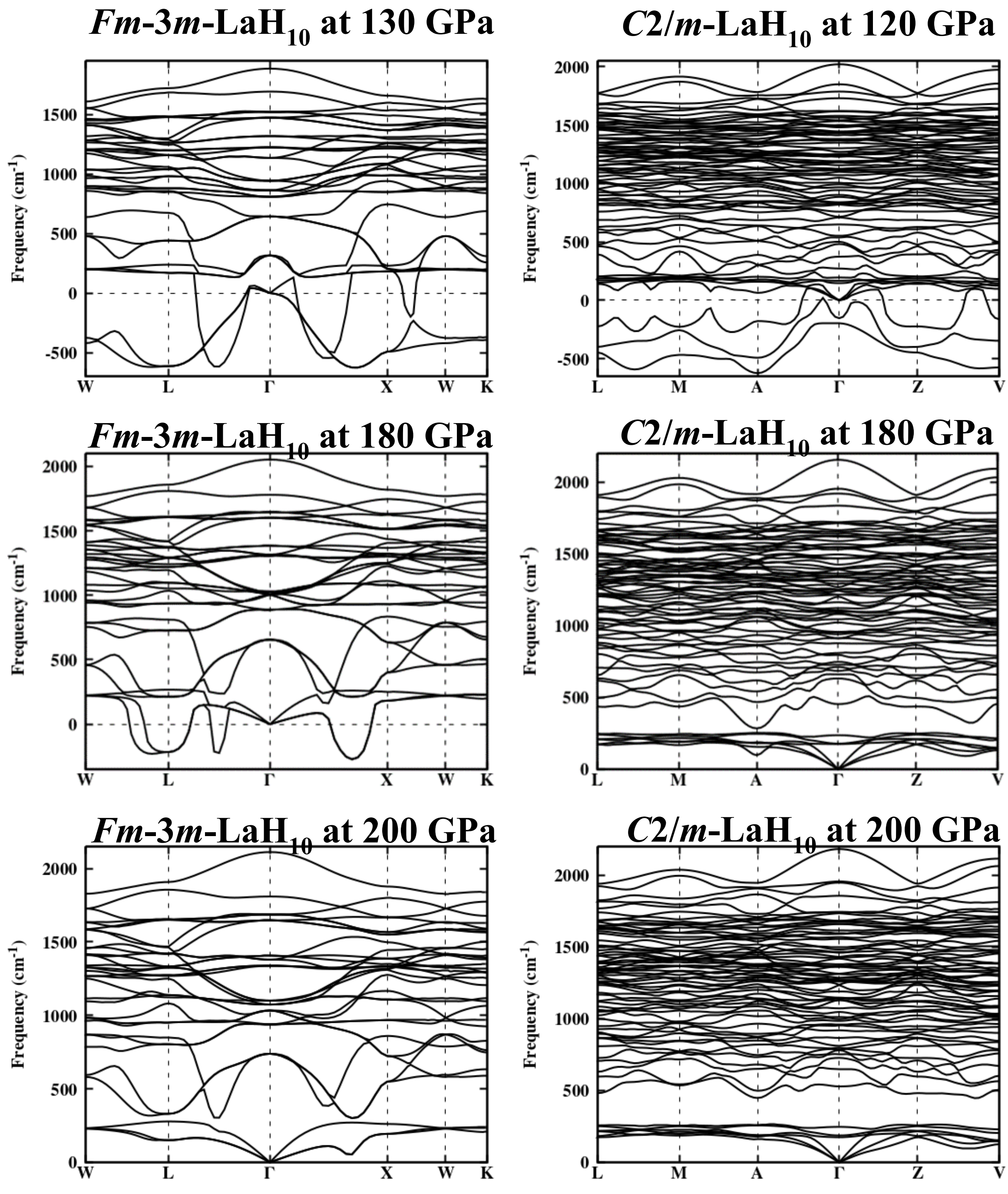


**Supplementary Fig. 4 Calculated phonon dispersions.** Harmonic phonon dispersion for the *Fm*-3*m* and *C*2/*m* phases of $LaH_{10}$ was calculated at several different values of pressure. We observe a trend towards the softening of certain phonons for the *Fm*-3*m* phase, especially along the Γ-X direction, that makes the structure unstable upon decompression and leads to the monoclinic *C*2/*m* distortion.

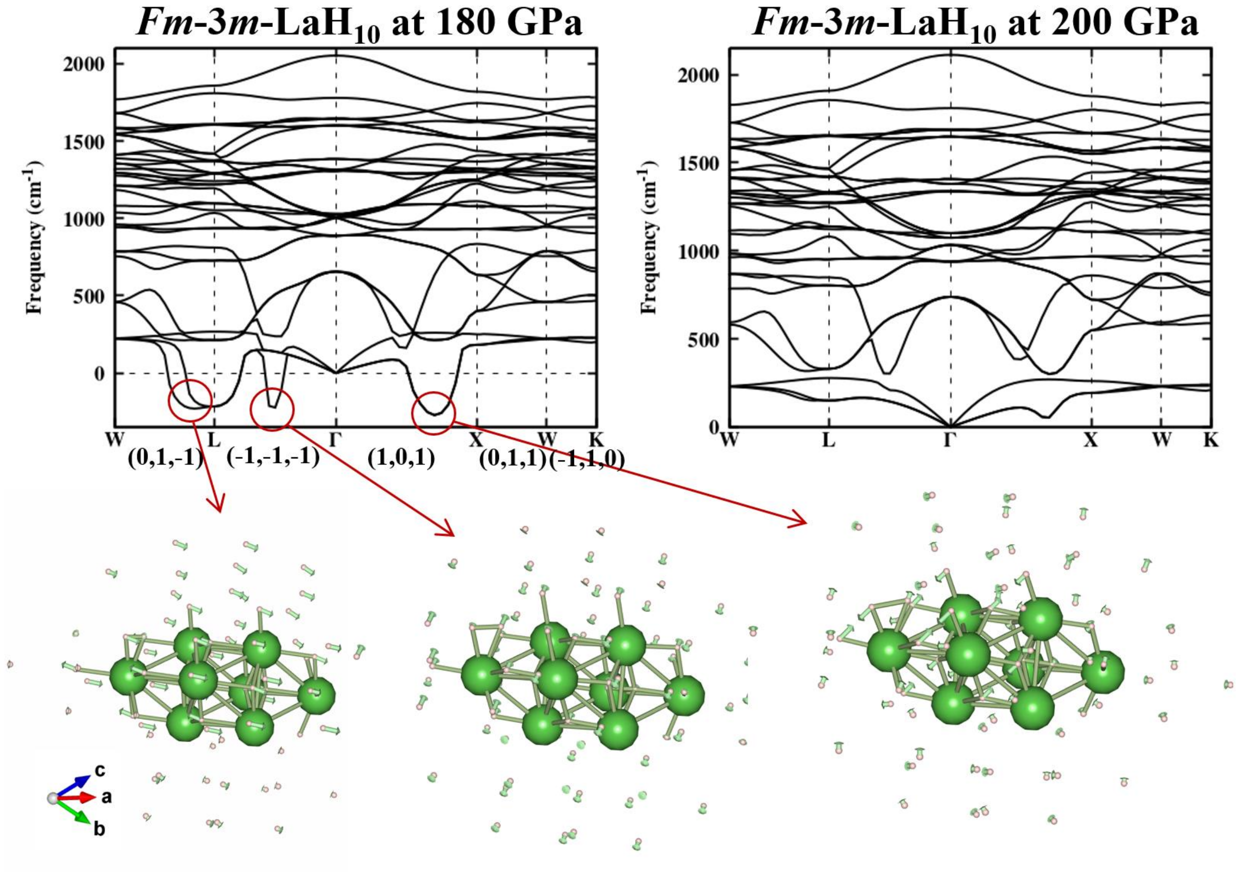


**Supplementary Fig. 5. Visualization of the atomic vibrations for three soft phonon modes in the *Fm-3m*-$LaH_{10}$ at 180 GPa.** Green and pink spheres represent La and H atoms, respectively. Green vectors indicate the motion for each atom. We find that freezing of the low-lying H-H "wagging" vibrations of the transverse acoustic phonon along the Γ-X direction (rightmost cartoon) leads to a structural instability and monoclinic *C2/m* distortion. The initial softening of the same phonon mode is obvious in the phonon spectrum at 200 GPa.

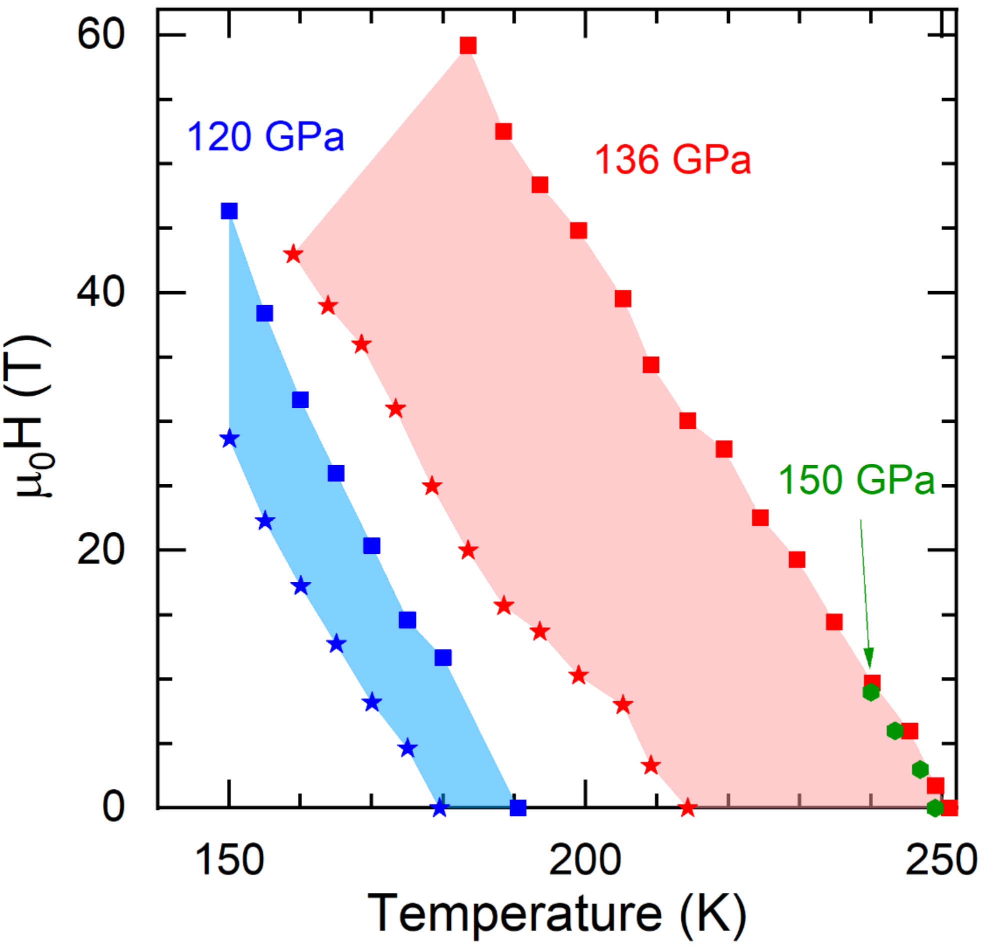


**Supplementary Fig. 6. The upper critical field $H_{c2}$ and the vortex melting field $H$* in $LaH_{10}$.** $H$* is estimated by extrapolating the leading edge of the transition to horizontal axis and $H_{c2}$ at the offset from the normal state magnetoresistance in $R(H)$ traces from Fig. 3. Stars denote the loci of $H$*, and squares denote that of $H_{c2}$. The blue and red symbols are for $LaH_{10}$ at 120 GPa and 136 GPa, respectively. The green hexagons are the $H_{c2}$ from reference[3]. The region of resistive dissipation between $H$* and $H_{c2}$ is the so-called vortex liquid state, denoted with red and blue shades. The measurement at 120 GPa has a narrower vortex liquid region while this region becomes wide after pressure is increased to 136 GPa. The upper critical field $H_{c2}$ of the 136 GPa measurement agrees well with the previous measurement up to 9 T in a sample with a similar $T_c$[3].

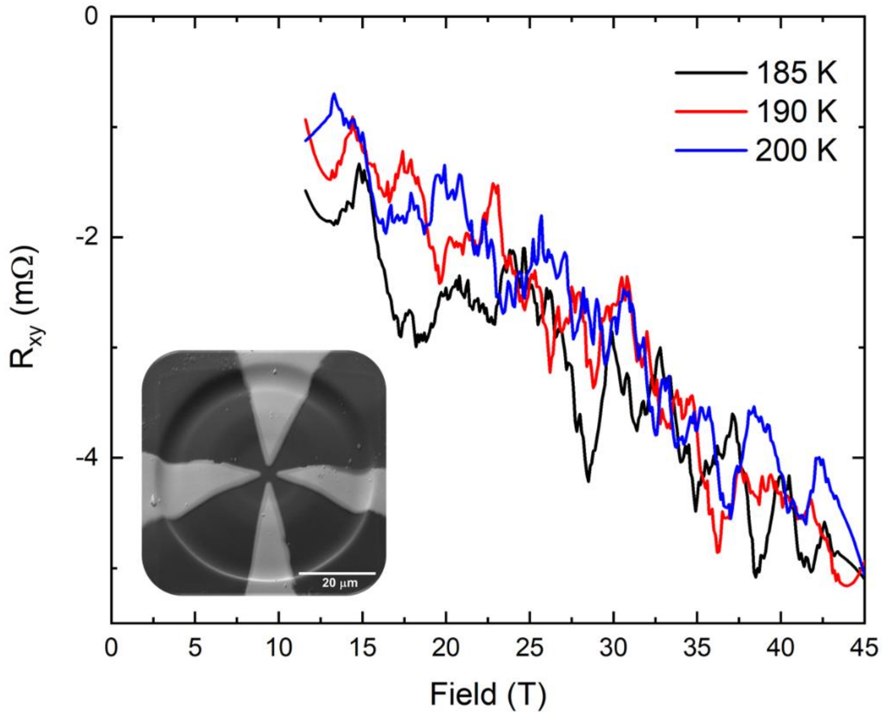


**Supplementary Fig. 7**. **The Hall resistance in the $LaH_{10}$ sample at 120 GPa.** The Hall effect is measured above $T_c$ in a DC magnet by sweeping the resistive insert field between 11.5 T and 45 T. $R_{xy}$ is linear at all three temperatures, which indicates that the magnetotransport in $LaH_{10}$ above $T_c$ is dominated by a single electron band. The Hall coefficient $R_H$ value is obtained by taking the slope of $R_{xy}$ and multiplying it by the thickness of the sample. The three measurements yield values ~ $2.3 \times 10^{-10} m^3/C$. Compared with $0.7 \times 10^{-10} m^3/C$ for $H_3S$[1], the $R_H$ value is 3 times larger for $LaH_{10}$. From a simple single band model, the density of electrons is $n = -1/R_H e = 7.2 \times 10^{21} cm^{-3}$. This value would indicate that $LaH_{10}$ has lower carrier density than $H_3S$, if one disregards some uncertainty about the actual conductive path within the sample. The temperature variance at this temperature range is small, although the variance may come from the noise in the measurement. We find that the Hall signal is lower than the noise in data collected under pulsed-fields at a pressure of 136 GPa, and thus it is not presented here. Inserts: a photo of the four sputtered electrical leads onto the diamond surface in a van der Pauw configuration.

# References

1. Mozaffari, S., Sun, D., Minkov, V., Drozdov, A., Knyazev, D., Betts, J., Einaga, M., Shimizu, K., Eremets, M., Balicas L.,& Balakirev, F. Superconducting phase diagram of $H_3S$ under high magnetic fields. *Nat. Commun.* **10**, 2522 (2019).

2. Hirsch, J. E., & Marsiglio, F. Intrinsic hysteresis in the presumed superconducting transition of hydrides under high pressure. *arXiv preprint arXiv:2101.07208* (2021).

3. Drozdov, A. P. et al. Superconductivity at 250 K in lanthanum hydride under high pressures. *Nature* **569**, 528-531 (2019).

4. Eremets, M. I. & Troyan, I. A. Conductive dense hydrogen. *Nat. Mater.* **10**, 927-931 (2011).

5. Semenok, DV. et al. Superconductivity at 253 K in lanthanum–yttrium ternary hydrides. *Materials Today* (2021).

6. Geballe, Z. M. et al. Synthesis and stability of lanthanum superhydrides. *Angew. Chem*. **130**, 696-700 (2018).

7. Eremets, M. I. Megabar high-pressure cells for Raman measurements. *J. Raman Spectrosc.* **34**, 515–518 (2003).
